# Loss of CDX2 in colorectal cancer is associated with histopathologic subtypes and microsatellite instability but is prognostically inferior to hematoxylin–eosin-based morphologic parameters from the WHO classification

**DOI:** 10.1038/s41416-021-01553-0

**Published:** 2021-10-06

**Authors:** Björn Konukiewitz, Maxime Schmitt, Miguel Silva, Junika Pohl, Corinna Lang, Katja Steiger, Kathrin Halfter, Jutta Engel, Anna Melissa Schlitter, Melanie Boxberg, Nicole Pfarr, Dirk Wilhelm, Sebastian Foersch, Markus Tschurtschenthaler, Wilko Weichert, Moritz Jesinghaus

**Affiliations:** 1 Institute of Pathology, Technical University Munich, Munich, Germany; 2 Institute of Pathology, Christian-Albrecht University of Kiel, Kiel, Germany; 3 II Medizinische Klinik, Klinikum rechts der Isar, Technical University Munich, Munich, Germany; 4 Munich Cancer Registry (MCR), at the University Hospital of Munich, Institute for Medical Information Processing, Biometry, and Epidemiology (IBE), Ludwig-Maximilian-University (LMU), Munich, Germany; 5 Department of Surgery, Klinikum rechts der Isar, Technical University Munich, Munich, Germany; 6 Institute of Pathology, University Hospital Mainz, Mainz, Germany; 7 Institute for Translational Cancer Research, German Cancer Consortium (DKTK), Partner Site Munich, Munich, Germany; 8 German Cancer Consortium (DKTK), Partner Site Munich, Munich, Germany; 9 Bavarian Cancer Center (BZKF), Munich, Germany; 10 Institute of Pathology, University Hospital Marburg, Marburg, Germany

**Keywords:** Biomarkers, Prognostic markers

## Abstract

**Background:**

Immunohistochemical loss of CDX2 has been proposed as a biomarker of dismal survival in colorectal carcinoma (CRC), especially in UICC Stage II/III. However, it remains unclear, how CDX2 expression is related to central hematoxylin–eosin (HE)-based morphologic parameters defined by 2019 WHO classification and how its prognostic relevance is compared to these parameters.

**Methods:**

We evaluated CDX2 expression in 1003 CRCs and explored its prognostic relevance compared to CRC subtypes, tumour budding and WHO grade in the overall cohort and in specific subgroups.

**Results:**

CDX2-low/absent CRCs were enriched in specific morphologic subtypes, right-sided and microsatellite-instable (MSI-H) CRCs (*P* < 0.001) and showed worse survival characteristics in the overall cohort/UICC Stage II/III (e.g. DFS: *P* = 0.005) and in microsatellite stable and left-sided CRCs, but not in MSI-H or right-sided CRCs. Compared with CDX2, all HE-based markers showed a significantly better prognostic discrimination in all scenarios. In multivariate analyses including all morphologic parameters, CDX2 was not an independent prognostic factor.

**Conclusion:**

CDX2 loss has some prognostic impact in univariate analyses, but its prognostic relevance is considerably lower compared to central HE-based morphologic parameters defined by the WHO classification and vanishes in multivariate analyses incorporating these factors.

## Introduction

Colorectal carcinoma (CRC) is a major cause of cancer-related deaths worldwide and is currently the third most common cancer among women and men in the United States [1, 2]. Histologically, CRC is a heterogeneous tumour entity. Besides the conventional adenocarcinoma NOS, a variety of histopathological CRC subtypes are known [3–12], which are usually given in routine pathology reports alongside other well-established morphological parameters, such as tumour budding or WHO grade. CRC subtypes, tumour budding and WHO grade are (mainly) assessed through the evaluation of hematoxylin–eosin (HE)-stained slides and are listed as essential diagnostic parameters for CRC in the WHO classification of tumours of the digestive system manual (WHO classification) [13]. Recently, we demonstrated the high prognostic relevance of these morphologic parameters provided by the recent WHO classification (CRC subtypes, tumour budding, WHO grade) in a large cohort of >1000 CRCs [8].

Caudal-related homeobox transcription factor 2 (CDX2) is a highly conserved master transcription factor that controls the development and differentiation of the intestinal epithelium [14, 15]. CDX2 is normally assessed via immunohistochemistry (IHC) with CDX2 being diffusely expressed in non-neoplastic colorectal mucosa and in the majority of CRCs [16]. Loss of CDX2 expression has been proposed as a biomarker of dismal clinical outcomes in CRC. Dalerba et al. reported that CDX2 loss might not only identify CRC patients with poorer survival characteristics but also those with an especially aggressive disease course in specific stage groups (e.g. UICC Stage II/III) [17]. While the general impact on survival of CDX2 loss has been confirmed by additional studies [18–26], it remains to be seen how CDX2 expression is related to the aforementioned HE-based morphologic factors and how the prognostic relevance of CDX2 ranks compared to these central morphologic parameters.

In order to address these questions, we evaluated CDX2 expression in a large cohort of over 1000 CRCs and analysed its connection to tumour budding, WHO grade and the various histomorphological CRC subtypes and compared the impact of all factors in the overall cohort, specific stage groups, right- vs. left-sided tumours as well as in microsatellite subgroups and finally in multivariate analyses incorporating all factors.

## Patients and methods

### Cohort

The investigated cohort comprised 1003 CRC patients that underwent surgical resection between 1997 and 2018 at the University Hospital Klinikum Rechts der Isar of the Technical University of Munich, Germany. Patients signed a general informed consent protocol during admission to the hospital. All patients with colorectal carcinomas from this time span with fully available clinicopathological/survival data as well as full block tumour tissue and from which tumour tissue was present on the constructed tissue microarray were included in this study. Tumours with other neoplasms of the colorectal system (e.g. neuroendocrine tumours, non-epithelial tumours etc.) were excluded. One case of an undifferentiated carcinoma from the original cohort was also excluded to avoid statistical bias. The clinicopathological characteristics, as well as survival data for all patients, were extracted from hospital records and from the Munich Cancer Registry. For overall survival (OS), all recorded patient deaths were noted, while for disease-specific survival (DSS) only tumour-associated deaths were recorded as events. Loco-regional or distant recurrence was noted as an event for disease-free survival (DFS). Endpoints of all survival comparisons were either events or a loss of follow-up, in which case the patients were censored at the time of the last available entry regarding the specific patient either in hospital records or in the Munich Cancer Registry. The treatment concepts of included patients followed internal policies, which were based on the given German guidelines at the time of diagnosis, generally meaning that all patients were intended to receive stage-adapted treatment. The definitive therapy regimen for each individual was decided by a multidisciplinary team of physicians during a specialised gastrointestinal tumour board. In most cases of colon cancer, this meant primary resection in all cases and adjuvant therapy in UICC Stage III. For Stage UICC II, adjuvant chemotherapy was only administered in high-risk patients (pT3/4, G3, < 70 years). For rectal cancers, neoadjuvant RCTx served as the standard for advanced cases (uT3N+) of the middle or lower third of the rectum, while non-advanced cancers and tumours of the proximal third of the rectum generally received primary surgery, followed by adjuvant chemotherapy depending on the post-operative tumour stage. The local ethics committee of the Technical University of Munich approved this study (reference number: 252/16s).

### Evaluation of CDX2 expression

CDX2 expression was evaluated in 1003 CRCs via CDX2 immunohistochemistry using a tissue microarray harbouring two tumour-carrying cores from each carcinoma. An automated immunostainer (BOND RXm System, Leica Biosystems, Germany) was used for immunohistochemical staining of CDX2 (clone: EPR2764Y, dilution: 1:500, Abcam, UK) on 2-µm sections from a tissue microarray. Briefly, after deparaffinization, antigen retrieval was performed with Epitope Retrieval 1 (Leica Biosystems, Germany; equivalent to citrate buffer pH6) for 20 min and antibody binding was detected using a Polymer Refine Detection Kit (Leica Biosystems, Germany) without post-primary antibody and hematoxylin counterstain. Appropriate positive and negative controls were run in parallel. The evaluation of CDX2 expression was performed manually by two experienced GI pathologists (MJ, BK) blinded to clinicopathological parameters.

Nuclear staining of CDX2 was considered specific. For each tumour, the number of positive tumour cells was counted and a cumulative percentage score for both cores was assigned for each CRC (range: 0–100%), for each tumour a minimum of 500 tumour cells were counted. The pattern of CDX2 expression was also analysed and divided into three classes: absent if no staining was visible, heterogeneous if areas with a complete loss of staining were noted and diffuse, if the tumours either showed a complete expression in 100% of the tumour cells or only a CDX2 loss in intermingled cells. Using normal colorectal mucosa as a reference, a staining intensity comparable to normal mucosa was considered as strong, a slightly weaker but still clearly visible staining was classified as medium and a faint, barely visible staining intensity was considered as weak. When no expression was detectable, the staining intensity was classified as absent.

### Evaluation of HE-based morphologic parameters

After we screened the CRCs included in our cohort for their expression of CDX2, we aimed to correlate their CDX2 expression status with H&E-based histopathological parameters. All of the tumours that were investigated for CDX2 expression in this study (*n* = 1003), received an in-depth histopathological characterisation in a previous study from our group, where this cohort was analysed by two expert GI pathologists (MJ, WW) regarding the distribution and the prevalence of the essential morphologic criteria given in the 2019 WHO classification of colorectal carcinoma [8]. As described previously in detail, full block H&E slides from CRC resection specimens were re-classified according to the current CRC subtypes listed in the 2019 WHO classification (adenocarcinoma NOS, mucinous adenocarcinoma, signet-ring carcinoma, medullary carcinoma, serrated adenocarcinoma, micropapillary adenocarcinoma, adenoma-like adenocarcinoma, adenosquamous carcinoma, carcinoma with sarcomatoid components, undifferentiated carcinoma, MANEC/NEC). Furthermore, all CRCs were analysed regarding their WHO grade (low grade, formerly G1/G2 vs. high grade, formerly G3) [13] and their tumour budding activity (Bd1: low tumour budding 0–4 buds in ×20 magnification, Bd2: intermediate tumour budding 5–9 buds in ×20 magnification, Bd3: high tumour budding >10 buds in ×20 magnification) [27]. Microsatellite status was available for all CRCs (assessed via IHC/PCR in our previous study) as described previously [8] (cohort details; Table 1).

**Table 1:** Distribution and prognostic impact of CDX2 expression, staging parameters as well as CRC subtypes, tumour budding and WHO grade in the overall cohort.

	Overall, *n* (%)	Median overall survival (SE)	*P* value	Mean disease-specific survival (SE)	*P* value	Mean disease-free survival (SE)	*P* value
Age			**<0.001**		**0.01**		**0.68**
Below median	*492 (49%)*	146.1 (5.4)		163.9 (4.9)		149.5 (5.1)	
Above median	*511 (51%)*	101.1 (4.8)		133.3 (4.7)		135.9 (4.6)	
Sex				**0.39**		**0.79**	**0.71**
Male	*575 (57.3%)*	125.5 (5.1)		157.6 (4.7)		151.9 (4.8)	
Female	*428 (42.7%)*	122.1 (5.1)		138.8 (4.9)		133.1 (5.0)	
CDX2 subgroups				**0.017**		**0.006**	**0.005**
CDX2-low/absent	*102 (10.2%)*	100.38 (10.3)		121.9 (10.7)		114.9 (10.7)	
CDX2-high	*901 (89.8%)*	131.28 (3.8)		158.7 (3.8)		153.1 (3.8)	
pT			**<0.001**		**<0.001**		**<0.001**
1	*78 (7.8%)*	139.1 (8.4)		172.4 (4.8)		165.2 (6.2)	
2	*182 (18.1%)*	140.5 (6.6)		164.6 (5.9)		161.0 (6.0)	
3	*554 (55.3%)*	125.8 (5.3)		156.5 (5.0)		148.7 (5.1)	
4	*189 (18.8%)*	87.8 (7.3)		96.4 (7.6)		90.6 (3.7)	
pN			**<0.001**			**<0.001**	**<0.001**
0	*552 (55%)*	141.0 (4.5)		173.9 (4.1)		174.3 (4.0)	
1	*284 (28.3%)*	112.2 (5.6)		124.9 (5.6)		115.2 (5.8)	
2	*167(16.7%)*	83.4 (8.7)		91.9 (8.9)		67.4 (8.6)	
pM			**<0.001**		**<0.001**		**<0.001**
0	*851 (84.9%)*	142.4 (4.3)		174.0 (3.8)		169.1 (3.8)	
1	*152 (15.1%)*	48.7 (5.0)		53.2 (5.4)		41.5 (5.18)	
UICC stage			**<0.001**		**<0.001**		**<0.001**
1	*207 (20.6%)*	147.4 (5.9)		178.9 (4.5)		175.9 (4.7)	
2	*326 (32.5%)*	133.6 (6.0)		164.0 (6.5)		167.8 (5.5)	
3	*318 (31.8%)*	130.2 (7.5)		153.8 (6.5)		136.9 (6.6)	
4	*152 (15.1%)*	48.3 (5.0)		52.8 (3.6)		41.5 (5.1)	
Tumour type (WHO)			**<0.001**		**<0.001**		**<0.001**
Adenocarcinoma NOS	*630 (62.8%)*	137.2 (3.9)		164.7 (4.4)		159.7 (4.4)	
Mucinous adenocarcinoma	*83 (8.3%)*	100.4 (9.0)		118.2 (9.4)		109.2 (9.7)	
Signet-ring cell carcinoma	*9 (0.9%)*	65.5 (28.3)		65.4 (28.3)		40.2 (23.8)	
Medullary adenocarcinoma	*32 (3.2%)*	147.9 (13.5)		188.0 (6.5)		181.5 (8.9)	
Micropapillary adenocarcinoma	*122 (12.2%)*	79.1 (9.0)		88.4 (9.7)		76.6 (9.5)	
Serrated adenocarcinoma	*87 (8.6%)*	119.6 (10.4)		141.7 (10.0)		133.7 (5.5)	
Adenoma-like adenocarcinoma	*33 (3.3%)*	132.0 (12.3)		170.6 (7.9)		176.8 (5.5)	
MANEC/NEC	*7 (0.7%)*	18.0 (8.1)		18.0 (8.1)		17.5 (9.3)	
WHO grade			**<0.001**		**<0.001**		**<0.001**
Low grade	*685 (68%)*	135.3 (4.3)		162.7 (4.0)		155.8 (4.0)	
High grade	*318 (32%)*	104.2 (6.3)		125.7 (6.4)		120.6 (6.6)	
Tumour budding			**<0.001**		**<0.001**		**<0.001**
Bd1	*545 (54%)*	160.9 (4.4)		192.3 (3.4)		190.6 (3.3)	
Bd2	*261 (26%)*	108.6 (8.0)		131.1 (7.2)		113.8 (7.2)	
Bd3	*197 (20%)*	48.5 (4.6)		56.2 (5.4)		48.5 (5.3)	
Resection margin			**<0.001**		**<0.001**		**<0.001**
R0	*932 (92.9%)*	135.8 (4.1)		166.8 (3.7)		160.2 (3.7)	
R1	*41 (4.1%)*	50.9 (10.5)		53.6 (11.0)		36.9 (9.5)	
R2	*30 (3%)*	26.1 (5.4)		26 (5.4)		21.5 (3.7)	
Lymphatic invasion			**<0.001**		**<0.001**		**<0.001**
Not present	*495 (49.3%)*	141.2 (4.8)		174.9 (4.3)		175.8 (4.2)	
Present	*508 (50.7%)*	111.1 (5.7)		129.9 (5.3)		116.2 (5.3)	
Venous invasion			**<0.001**		**<0.001**		**<0.001**
Not present	*870 (86.8%)*	136.2 (4.2)		166.8 (3.7)		162.8 (3.8)	
Present	*133 (13.2%)*	74.9 (9.1)		79.06 (9.6)		63.3 (8.6)	
Perineural invasion			**<0.001**		**<0.001**		**<0.001**
Not present	*936 (93.3%)*	132.6 (4.1)		162.1 (3.7)		158.0 (3.7)	
Present	*67 (6.7%)*	50.4 (6.1)		50.4 (6.1)		35.1 (5.4)	
Microsatellite status			**0.01**		**0.01**		**0.01**
Microsatellite stable	*849 (84.6%)*	125.5 (4.2)		151.68 (4.0)		145.3 (4.0)	
Microsatellite instable	*154 (15.4%)*	137.0 (8.0)		165.7 (7.2)		162.8 (7.2)	
Tumour localisation			**0.18**		**0.38**		**0.33**
Caecum	*147 (14.7%)*	109.2 (6.9)		130.0 (6.2)		128.9 (6.8)	
Ascending colon	*256 (25.5%)*	120.5 (6.3)		143.2 (6.8)		132.9 (6.4)	
Transverse colon	*82 (8.2%)*	89.0 (8.6)		110.1 (9.1)		112.1 (9.4)	
Descending colon	*92 (9.2%)*	128.1 (11.7)		143.3 (11.9)		143.1 (11.8)	
Sigmoid colon	*318 (31.7%)*	136.6 (6.7)		162.4 (6.2)		156.2 (6.3)	
Rectum	*108 (10.8%)*	103.9 (8.6)		119.9 (9.2)		110.2 (9.4)	

Bold values indicate statistical significance.

### Statistics

Statistical analyses were performed using SPSS version 26 (SPSS Institute, Chicago, IL) using *Χ*^2^ test as well as *Χ*^2^ test for trends and Fisher’s exact test. The Bonferroni method was used to correct for multiple testing. Survival comparisons were performed using the Kaplan–Meier method and a log-rank test was used to test the significance of survival differences. Multivariate analyses were done with the Cox proportional hazard model. All statistical tests were performed two-sided, *P* values ≤0.05 were considered significant.

## Results

### Clinicopathological characteristics

Fifty-seven percent (*n* = 575) of the 1003 patients were male, the median patient age was 69 years. Right-sided (caecum until splenic flexure; *n* = 485; 48%) and left-sided CRCs (descending colon until rectum; *n* = 518; 52%) were almost evenly present. Using the eighth edition of the TNM classification of malignant tumours [28], pTNM staging was determined (207 Stage I (21%), 326 (32%) Stage II, 318 (32%) Stage III and 152 (15%) Stage IV tumours). Three hundred and twenty-two patients relapsed (32%), 408 patients (40.7%) died during follow-up, for 293 patients a tumour specific death was noted (cohort details: Table 1).

### Distribution of CDX2 expression and CDX2 groups

Most CRCs showed a diffuse CDX2 expression (median: 100% positive tumour cells, mean: 91% positive tumour cells) resulting in 783 CRCs (78%) showing a diffuse nuclear expression in all tumours cells and a total of 85% (852/1003) showing a diffuse nuclear staining in ≥90% of tumour cells. Forty-seven (5%) CRCs showed a nuclear expression in 61–89% of tumour cells and 90 carcinomas (9%) showed a reduced CDX2 staining within the range of 1–60% positive tumour cells. A complete loss of CDX2 expression was noted for 12 CRCs (1%). In order to perform deeper statistical analyses, we needed to find an ideal cut-off to form dichotomous CDX2 groups comparable to previous studies, which was guided using the Cutoff Finder [29]. Afterwards, CRCs were assigned into two groups regarding their CDX2 expression (CDX2 groups): CRCs that showed a CRC expression that was on the 10th percentile and below (range: 0–60% of tumour cells; *n* = 102, 10%) were categorised as CDX2-low/absent and CRCs with a CDX2 expression above the 10th percentile (61–100% tumour cells; *n* = 901, 90%) were categorised as CDX2- high. CDX2-low/absent CRCs usually showed a reduced CDX2 staining intensity (CDX2-low/absent: strong staining intensity: 0/102 (0%), medium staining intensity: 24/102 (23%), weak staining intensity: 66/102 (65%), absent staining: 12/102 (12%); CDX2-high: strong staining intensity: 842/901 (94%), medium staining intensity: 59/901 (6%), *P* < 0.001) and a significantly higher rate of a heterogeneous/absent staining pattern when compared to CDX2-high tumours (CDX2-low/absent: absent: 12/102 (12%), heterogeneous: 90/102 (88%); CDX2-high: heterogeneous: 26/901 (3%), diffuse: 875/901 (97%), *P* < 0.001). A comparison of the results of the CDX2 grouping from 20 randomly selected full block slides showed a 100% concordance with the results from the TMA (*P* < 0.001). Furthermore, the interobserver variance between the two observers was assessed in 150 randomly selected cases, where an excellent correlation for the respective CDX2 groups was evident (*P* < 0.001, Kappa–Cohens value: 0.84).

### Distribution of morphologic parameters (CRC subtypes/tumour budding/WHO grade) and microsatellite status

CRC subtypes were present in the following numbers: 630 adenocarcinomas NOS (63%), 122 micropapillary adenocarcinomas (12%), 87 serrated adenocarcinomas (9%), 83 mucinous (8%) adenocarcinomas, 33 adenoma-like adenocarcinomas 3%), 32 medullary carcinomas (3%), 9 signet-ring cell carcinomas (1%), 7 (1%) mixed adenoneuroendocrine carcinomas (MANEC)/neuroendocrine carcinomas (NEC). A low tumour budding activity (Bd1) was observed in 545 CRCs (54%), intermediate tumour budding (Bd2) was detected in 261 tumours (26%), a high tumour budding activity (Bd3) was observed in 197 cases (20%). Six hundred and eighty-five CRCs were graded as low grade (68%) and 318 as high grade (32%) according to the WHO-grading system. Eight-hundred and forty-nine CRCs were microsatellite stable (MSS; 849/1003, 85%) and 154 were classified as microsatellite instable (MSI-High: 154/1003, 15%, details Table 1).

### Association of CDX2 groups with tumour stage, morphologic parameters (CRC subtypes/tumour budding/WHO grade) and microsatellite status

**Fig. 1:**
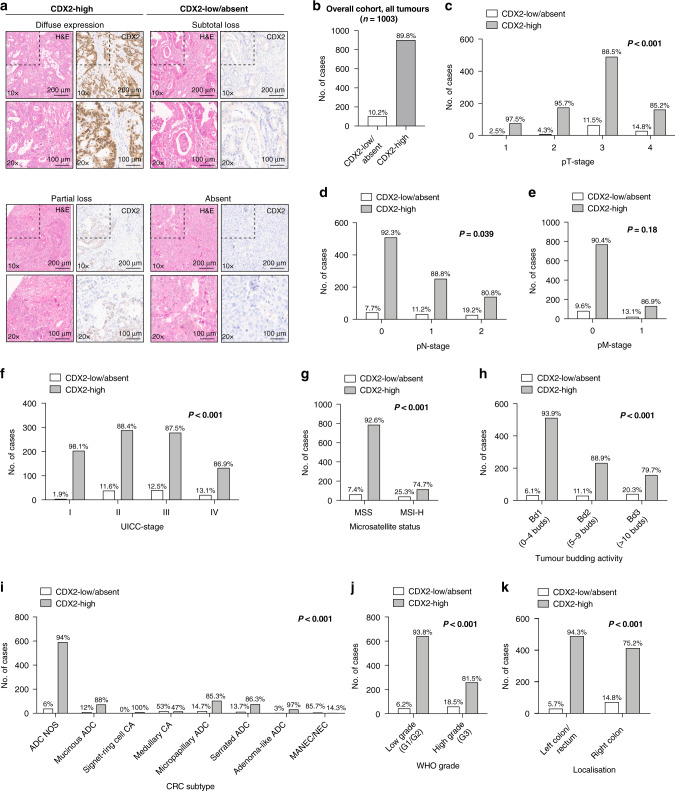
Histopathology of CDX2-expression groups and distribution of CDX2 groups with HE-based morphological factors and clinicopathological parameters. Histopathology (**a**) and frequency of CDX2-expression groups (**b**) as well as the association of CDX2-expression groups with pTNM status (**c**–**e**), UICC stage (**f**) and microsatellite status (**g**) in the overall cohort. Further relationship of CDX2-expression groups with tumour budding subgroups (**h**), CRC subtypes (**i**) and WHO grade (**j**) as well as tumour localisation (**k**) in the overall cohort. ADC   adenocarcinoma, CA   carcinoma.

As depicted in detail in Fig. 1 and Table 2, CDX2-low/absent CRCs were significantly more frequent in higher pT/pN/UICC-stages (*P* < 0.001, *P* = 0.039, *P* < 0.001, respectively) and right-sided tumours (*P* < 0.001). Compared to CDX2-high neoplasms, CDX2-low/absent CRCs were also more frequent in high-grade CRCs according to the WHO grade and tumours with intermediate (Bd2) or high (Bd3) tumour budding activity as well as in MSI-H CRCs (*P* < 0.001, respectively). However, the majority of cases with either increased tumour budding activity or high WHO grade fell into the CDX2-high category.

**Table 2:** Association of CDX2 expression with clinicopathological parameters as well as CRC subtypes, tumour budding and WHO grade in the overall cohort.

	Overall	CDX2-low/absent	CDX2-high	*P* value
	*1003 (100%)*	102 (10.2%)	901 (89.8%)	
Age				**0.002**
Below median	492 (49%)	35 (7.1%)	457 (92.9%)	
Above median	511 (51%)	67 (13.1%)	444 (86.9%)	
Gender				**0.001**
Female	428 (42.7%)	59 (13.8%)	369 (86.2%)	
Male	575 (57.3%)	43 (7.5%)	532 (92.5%)	
pT				**<0.001**
1	78 (7.8%)	2 (2.6%)	76 (97.4)	
2	182 (18.1%)	8 (4.4%)	174 (95.6%)	
3	554 (55.2%)	64 (11.6%)	490 (88.4%)	
4	189 (18.8%)	28 (14.8%)	161 (85.2%)	
pN				**0.039**
0	552 (55%)	43 (7.8%)	509 (92.2%)	
1	284 (28.3%)	32 (11.3%)	252 (88.7%)	
2	167 (16.7%)	27 (16.2%)	140 (83.8%)	
pM				**0.18**
0	851 (84.8%)	82 (9.6%)	769 (90.4%)	
1	152 (15.2%)	20 (13.2%)	132 (86.8%)	
UICC stage				**<0.001**
1	207 (20.6%)	4 (1.9%)	203 (98.1%)	
2	326 (32.5%)	38 (11.7%)	288 (88.3%)	
3	318 (31.7%)	40 (12.6%)	278 (87.4%)	
4	152 (15.2%)	20 (13.2%)	132 (86.8%)	
Tumour type (WHO)				**<0.001**
Adenocarcinoma NOS	630 (62.8%)	38 (6%)	592 (94%)	
Mucinous adenocarcinoma	83 (8.3%)	10 (12%)	73 (88%)	
Signet-ring cell carcinoma	9 (0.9%)	0 (0%)	9 (100%)	
Medullary adenocarcinoma	32 (3.2%)	17 (53.1%)	15 (46.9%)	
Micropapillary adenocarcinoma	122 (12.2%)	18 (14.7%)	104 (85.3%)	
Serrated adenocarcinoma	87 (8.7%)	12 (13.8%)	75 (86.2%)	
Adenoma-like adenocarcinoma	33 (3.3%)	1 (3%)	32 (97%)	
MANEC/NEC	7 (0.7%)	6 (85.7%)	1 (14.3%)	
WHO grade				**<0.001**
Low grade	685 (68.3%)	43 (6.3%)	642 (93.7%)	
High grade	318 (31.7%)	59 (18.5%)	259 (81.5%)	
Tumour budding				**<0.001**
Bd1	545 (54.3%)	33 (6.1%)	512 (93.9%)	
Bd2	261 (26%)	29 (11.1%)	232 (88.9%)	
Bd3	197 (19.7%)	40 (20.3%)	157 (79.7%)	
Resection margin				**0.024**
R0	932 (92.9%)	91 (9.8%)	841 (90.2%)	
R1	41 (4.1%)	10 (24.4%)	31 (75.6%)	
R2	30 (3%)	1 (3.3%)	29 (96.7%)	
Lymphatic invasion				**0.001**
Not present	495 (49.4%)	34 (6.9%)	461 (93.1%)	
Present	508 (50.6%)	68 (13.4%)	440 (86.6%)	
Venous invasion				**0.003**
Not present	870 (86.7%)	81 (9.3%)	789 (90.7%)	
Present	133 (13.3%)	21 (15.8%)	112 (84.2%)	
Perineural invasion				**0.29**
Not present	936 (93.3%)	98 (10.5%)	838 (89.5%)	
Present	67 (6.7%)	4 (6%)	63 (94%)	
Microsatellite status				**<0.001**
Microsatellite stable	849 (84.6%)	63 (7.4%)	786 (92.6%)	
Microsatellite instable	154 (15.4%)	39 (25.3%)	115 (74.7%)	
Tumour localisation				**0.01**
Caecum	147 (14.7%)	22 (15%)	125 (85%)	
Ascendening colon	256 (25.5%)	43 (16.8%)	213 (83.2%)	
Transverse colon	82 (8.2%)	7 (8.5%)	75 (91.5%)	
Descending colon	92 (9.2%)	9 (9.8%)	83 (90.2%)	
Sigmoid colon	318 (31.7%)	13 (4.1%)	305 (95.9%)	
Rectum	108 (10.8%)	8 (7.4%)	100 (92.6%)	

Bold values indicate statistical significance.

The presence of CDX2-low/absent tumours in general was also significantly associated with the presence of certain histopathological CRC subtypes from both ends of the spectrum of biological aggressiveness (*P* < 0.001). For example, >50% of the prognostically favourable (and frequently microsatellite-instable) medullary carcinomas as well as almost all of the biologically highly aggressive MANEC/NECs fell into the CDX2-low/absent subgroup.

### Prognostic relevance of CDX2 groups in the overall cohort, microsatellite subgroups, right- vs. left-sided tumours and UICC Stage II/III CRCs

**Fig. 2:**
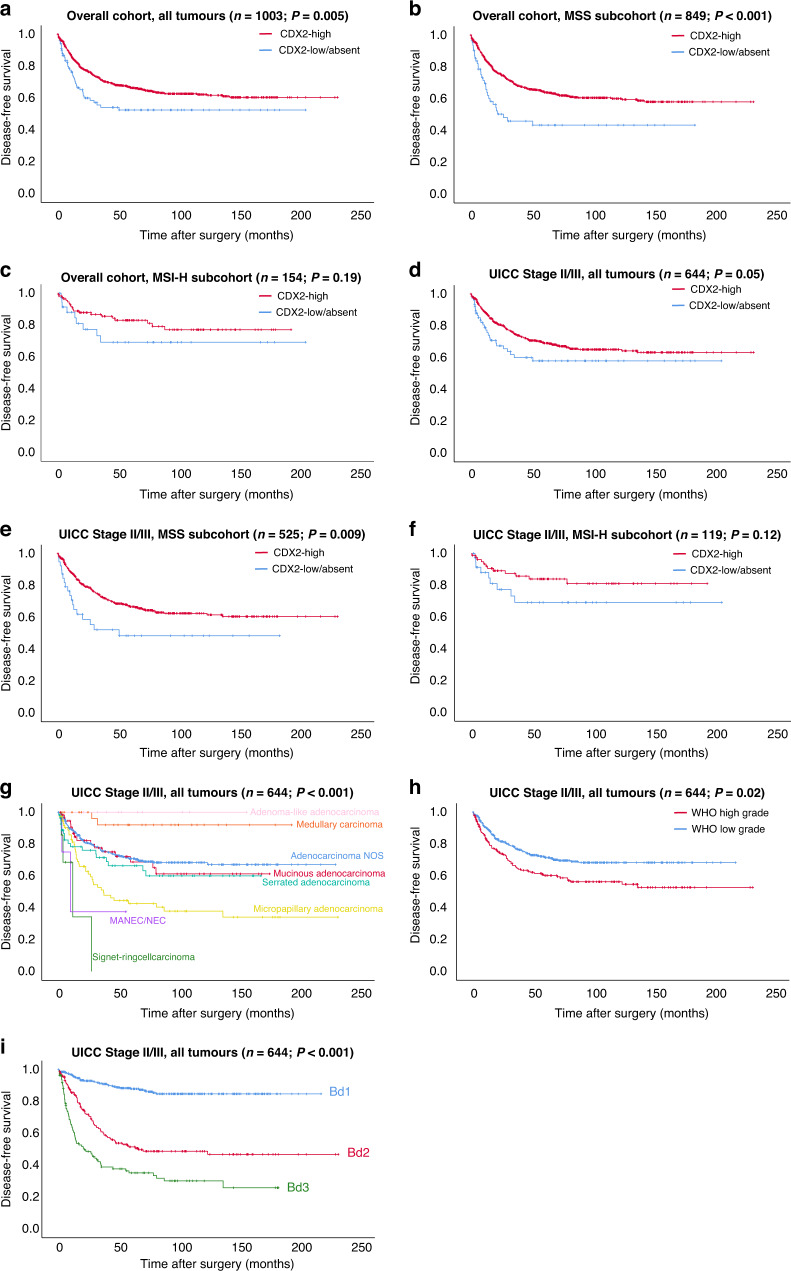
Prognostic relevance of CDX2 and HE-based morphologic factors. Prognostic relevance of CDX2 expression groups on disease-free survival in the overall cohort including microsatellite subgroups (**a**–**c**), UICC Stage II/III tumours including microsatellite subgroups (**d**–**f**) and of the central HE-based morphologic factors CRC subtypes, WHO grade and tumour budding in all UICC Stage II/III tumours (**g**–**i**).

As illustrated in Fig. 2, Supplementary Fig. 1 and Table 1, compared to CDX2-high tumours, the CDX2-low/absent group showed a decreased OS (CDX2-high 131.2 months vs CDX2-low/absent 100.3 months, *P* = 0.017), DSS (CDX2-high 158.7 months vs CDX2-low/absent 121.9 months, *P* = 0.006) and DFS (CDX2-high: 153.9 months vs CDX2-low/absent 114.9 months, *P* = 0.005) in the overall cohort of 1003 CRCs. As depicted in Table 3, the decreased survival of the CDX2-low/absent group was pronounced in the subgroup of MSS-CRCs (*n* = 849; OS: CDX2-high 127.4 months vs CDX2-low/absent 87.1 months, *P* = 0.012; DSS: CDX2-high 154.6 months vs CDX2-low/absent 94.7 months, *P* < 0.001; DFS: CDX2-high 148.2 months vs CDX2-low/absent 87.5 months, *P* < 0.001), but was not significant in the subgroup of MSI-high CRCs (*n* = 154; OS/DSS/DFS, *P* > 0.05).

**Table 3:** Prognostic impact of CDX2 expression and CRC subtypes, tumour budding and WHO grade in microsatellite subgroups of the overall cohort.

Overall cohort		Overall, *n* (%)	Mean overall survival (SE)	*P* value	Mean disease-specific survival (SE)	*P* value	Mean disease-free survival (SE)	*P* value
Microsatellite status: MSS subcohort				**0.012**		**<0.001**		**<0.001**
CDX2 expression	CDX2-low/absent	63 (7.4%)	87.19 (11.32)		94.71 (11.69)		87.56 (11.85)	
	CDX2-high	786 (92.6%)	127.48 (4.42)		154.65 (4.16)		148.30 (4.21)	
Microsatellite status: MSI-high subcohort				**0.083**		**0.223**		**0.198**
CDX2 expression	CDX2-low/absent	39 (25.3%)	114.33 (16.65)		157.33 (15.27)		148.10 (16.49)	
	CDX2-high	115 (74.7%)	138.31 (8.11)		160 (7.32)		157.83 (7.40)	
Microsatellite status: MSS subcohort				**<0.001**		**<0.001**		**<0.001**
Tumour budding activity	Bd1	435 (51.2%)	159.06 (5.04)		190.86 (3.96)		190.31 (3.78)	
	Bd2	231 (27.2%)	108.09 (7.60)		128.72 (7.68)		108.26 (7.64)	
	Bd3	183 (21.6%)	49.29 (4.84)		57.00 (5.64)		49.40 (5.59)	
Microsatellite status: MSI-high subcohort				**<0.001**		**<0.001**		**<0.001**
Tumour budding activity	Bd1	110 (71.4%)	151.11 (7.80)		177.36 (5.81)		171.86 (6.43)	
	Bd2	30 (19.5%)	108.11 (17.08)		137.11 (18.46)		147.09 (17.60)	
	Bd3	14 (9.1%)	36.22 (11.87)		32.71 (10.37)		24.76 (9.53)	
Microsatellite status: MSS subcohort				**<0.001**		**<0.001**		**<0.001**
WHO grade	Low grade	602 (70.9%)	132.70 (4.67)		160.66 (4.30)		152.86 (4.33)	
	High grade	247 (29.1%)	96.98 (6.69)		112.11 (7.20)		106.25 (7.39)	
Microsatellite status: MSI-high subcohort				**0.128**		**0.166**		**0.201**
WHO grade	Low grade	83 (53.9%)	149.00 (11.18)		171.27 (10.30)		170.35 (9.75)	
	High grade	71 (46.1%)	120.51 (10.42)		149.05 (9.88)		145.30 (10.23)	
Microsatellite status: MSS subcohort				**<0.001**		**<0.001**		**<0.001**
CRC subtypes	Adenocarcinoma NOS	562 (66.2%)	134.61 (5.24)		162.19 (4.74)		156.13 (4.79)	
	Mucinous adenocarcinoma	55 (6.5%)	94.01 (10.93)		111.43 (11.65)		101.17 (11.96)	
	Signet-ring cell carcinoma	6 (0.7%)	15.13 (7.49)		15.13 (7.49)		12.34 (6.23)	
	Medullary adenocarcinoma	4 (0.5%)	129.68 (24.43)		129.68 (24.43)		128.47 (22.01)	
	Micropapillary adenocarcinoma	117 (13.8%)	79.68 (9.19)		89.14 (9.92)		77.00 (9.75)	
	Serrated adenocarcinoma	73 (8.6%)	113.79 (11.33)		137.73 (11.13)		133.81 (11.49)	
	Adenoma-like adenocarcinoma	28 (3.2%)	138.34 (13.24)		175.78 (6.57)		167.94 (9.68)	
	MANEC/NEC	4 (0.5%)	9.23 (4.90)		9.23 (4.90)		8.07 (3.25)	
Microsatellite status: MSI-high subcohort				**0.338**		**0.007**		**0.019**
CRC subtypes	Adenocarcinoma NOS	68 (44.2%)	145.93 (11.85)		169.73 (10.73)		172.25 (10.00)	
	Mucinous adenocarcinoma	28 (18.2%)	111.37 (15.54)		128.41 (15.68)		123.49 (15.84)	
	Signet-ring cell carcinoma	3 (1.9%)	82.13 (46.93)		82.13 (46.93)		80.25 (48.26)	
	Medullary adenocarcinoma	28 (18.2%)	142.62 (14.86)		187.09 (7.40)		179.72 (10.08)	
	Micropapillary adenocarcinoma	5 (3.2%)	38.56 (14.61)		38.56 (14.61)		35.22 (16.06)	
	Serrated adenocarcinoma	14 (9.1%)	132.57 (18.59)		140.75 (17.87)		120.05 (20.30)	
	Adenoma-like adenocarcinoma	5 (3.2%)	98.87 (26.65)		139.70 (28.49)		143.46 (29.84)	
	MANEC/NEC	3 (1.9%)	38.46 (14.52)		38.46 (14.52)		38.46 (14.51)	

Bold values indicate statistical significance.

When analysed only in UICC Stage II/III CRCs (*n* = 644, Fig. 2, Supplementary Fig. 2 and Table 4), CDX2-low/absent CRCs had a slightly lower DFS (CDX2-high 157.8 months vs CDX2-low/absent 148.1 months, *P* = 0.05), but no significant differences were observed regarding OS/DSS (*P* > 0.05, respectively) between CDX2 groups. In microsatellite stable UICC Stage II/III CRCs, CDX2-low/absent tumours were associated with a significantly shorter DSS/DFS but not OS (*n* = 525; OS: *P* > 0.05; DSS: CDX2-high 160.7 months vs CDX2-low/absent 108.8 months, *P* = 0.05; DFS: CDX2-high 154.4 months vs CDX2-low/absent 96.8 months, *P* = 0.009), while in microsatellite-instable UICC Stage II/III CRCs, CDX2 expression had no impact on any survival parameter (OS/DSS/DFS, *P* > 0.05). When analysed in left- vs. right-sided CRCs, we observed a strong impact of CDX2 loss in univariate analyses of left-sided CRCs (*P* < 0.001 for all comparisons, Supplementary Fig. 3), which was also preserved in the subgroup of MSS left-sided CRCs (*P* < 0.001 for all comparisons) and MSI-H left-sided CRCs (e.g: DFS, *P* < 0.004). In right-sided CRCs, no prognostic effect of CDX2 was visible (*P* > 0.05 for all comparisons, data not shown).

**Table 4:** Prognostic impact of CDX2 expression and CRC subtypes, tumour budding and WHO grade in microsatellite subgroups of UICC Stage II/III CRCs.

UICC Stage II/III CRCs		Overall, *n* (%)	Mean overall survival (SE)	*P* value	Mean disease-specific survival (SE)	*P* value	Mean disease-free survival (SE)	*P* value
Microsatellite status: MSS subcohort				**0.261**		**0.05**		**0.009**
CDX2 expression	CDX2-low/absent	39 (7.4%)	100.53 (14.18)		108.11 (14.60)		96.84 (14.81)	
	CDX2-high	486 (92.6%)	133.55 (5.57)		160.71 (5.29)		154.04 (5.31)	
Microsatellite status: MSI-high subcohort				**0.069**		**0.170**		**0.125**
CDX2 expression	CDX2-low/absent	39 (32.8%)	114.33 (16.65)		157.33 (15.27)		148.10 (16.49)	
	CDX2-high	80 (67.2%)	140.79 (9.64)		164.17 (8.40)		162.96 (8.37)	
Microsatellite status: MSS subcohort				**<0.001**		**<0.001**		**<0.001**
Tumour budding activity	Bd1	265 (50.5%)	158.75 (6.60)		186.29 (5.56)		188.17 (4.99)	
	Bd2	160 (30.5%)	116.22 (8.96)		139.12 (9.16)		118.43 (9.27)	
	Bd3	100 (19%)	69.90 (7.50)		81.04 (8.54)		70.02 (8.45)	
Microsatellite status: MSI-high subcohort				**<0.001**		**<0.001**		**<0.001**
Tumour budding activity	Bd1	82 (68.9%)	153.20 (8.95)		182.55 (5.89)		177.94 (6.53)	
	Bd2	24 (20.2%)	110.77 (19.17)		146.80 (20.32)		148.86 (19.84)	
	Bd3	13 (10.9%)	38.85 (12.53)		32.52 (10.35)		26.74 (10.12)	
Microsatellite status: MSS subcohort				**0.021**		**0.001**		**<0.001**
WHO grade	Low grade	384 (73.1%)	132.22 (5.85)		160.30 (5.54)		154.36 (5.36)	
	High grade	141 (26.9%)	119.27 (9.16)		133.62 (9.48)		122.37 (9.77)	
Microsatellite status: MSI-high subcohort				**0.124**		**0.271**		**0.283**
WHO grade	Low grade	55 (46.2%)	153.56 (14.02)		175.68 (12.27)		176.71 (10.49)	
	High grade	64 (53.8%)	120.86 (10.93)		152.77 (10.20)		148.68 (10.62)	
Microsatellite status: MSS subcohort				**0.023**		**<0.001**		**<0.001**
CRC subtypes	Adenocarcinoma NOS	355 (67.6%)	138.24 (6.45)		164.18 (6.08)		161.02 (5.94)	
	Mucinous adenocarcinoma	36 (6.8%)	107.63 (13.25)		130.72 (13.39)		121.12 (14.16)	
	Signet-ring cell carcinoma	5 (0.9%)	21.09 (8.17)		21.09 (8.17)		16.37 (7.42)	
	Medullary adenocarcinoma	4 (0.8%)	128.47 (22.01)		128.47 (22.01)		128.47 (22.01)	
	Micropapillary adenocarcinoma	69 (13.1%)	109.10 (13.00)		122.12 (13.54)		102.33 (13.50)	
	Serrated adenocarcinoma	47 (8.9%)	96.96 (11.43)		115.32 (11.76)		100.91 (11.57)	
	Adenoma-like adenocarcinoma	8 (1.5%)	133.30 (23.63)		133.30 (16.71)		133.30 (16.71)	
	MANEC/NEC	1 (0.2%)	21.67 (0.00)		21.67 (0.00)		10.00 (0.00)	
Microsatellite status: MSI-high subcohort				**0.131**		**0.002**		**0.010**
CRC subtypes	Adenocarcinoma NOS	47 (39.5%)	142.68 (14.62)		169.78 (13.12)		172.86 (11.70)	
	Mucinous adenocarcinoma	23 (19.3%)	107.05 (15.77)		127.18 (15.82)		119.86 (16.63)	
	Signet-ring cell carcinoma	2 (1.7%)	16.77 (0.00)		15.77 (0.00)		12.00 (0.00)	
	Medullary adenocarcinoma	28 (23.5%)	142.62 (14.86)		187.09 (7.40)		179.72 (10.08)	
	Micropapillary adenocarcinoma	5 (4.2%)	38.56 (14.61)		38.56 (14.61)		35.22 (16.06)	
	Serrated adenocarcinoma	9 (7.6%)	148.35 (19.38)		150.43 (17.01)		146.09 (21.28)	
	Adenoma-like adenocarcinoma	2 (1.6%)	70.37 (0.00)		70.37 (0.00)		70.37 (0.00)	
	MANEC/NEC	3 (2.5%)	38.46 (14.51)		38.46 (14.51)		38.46 (14.51)	

Bold values indicate statistical significance.

When only adenocarcinomas NOS, the most common CRC subtype, were analysed, comparable survival results to the overall cohort were observed in all subgroups (e.g. DSS: *P* = 0.001 in all adenocarcinomas NOS, data not shown). No significant survival differences were observed between CDX2-high vs CDX2-low/absent tumours within the different tumour budding/WHO grade subgroups.

### Prognostic impact of HE-based morphologic parameters (CRC subtypes/tumour budding/WHO grade) in the overall cohort, microsatellite subgroups, right- vs. left-sided tumours and in UICC Stage II/III CRCs

Compared to the different CDX2 expression groups, the central HE-based morphologic parameters generally showed stronger survival discrimination than the CDX2-expression groups in the overall cohort and in Microsatellite subcohorts as well as UICC Stage 2/3 subgroups [8]. As depicted in detail in Table 1 and Supplementary Fig. 4, CRC subtypes strongly impacted on patient survival and showed a great variation regarding their OS/DSS/DFS (*P* < 0.001 for OS/DSS/DFS, respectively), with some subtypes like medullary carcinoma or adenoma-like adenocarcinoma showing a very indolent disease course and other specific variants like micropapillary adenocarcinoma or MANEC/NEC showing dismal survival characteristics. The different tumour budding categories (e.g. DFS: 190.6 months (Bd1) vs. 113.8 months (Bd2) vs. 48.5 (Bd3)) and the respective WHO-grades (e.g. DFS: 155.8 months (low grade) vs. 120.6 months (high grade)) were also strongly associated with patient survival on all survival comparisons (*P* < 0.001, respectively, for OS/DSS/DFS). When analysed separately in MSS- and MSI-H CRCs in the overall cohort, tumour budding (*P* < 0.001 for all comparisons in MSS/MSI-H) and CRC subtypes (DSS: *P* < 0.001 in MSS; *P* = 0.007 in MSI-H/DFS: *P* < 0.001 in MSS; *P* = 0.01 in MSI-H) retained their prognostic impact in both microsatellite subgroups, while comparable to CDX2, WHO grade only remained significant in MSS CRC (*P* < 0.001 for all comparisons in MSS; *P* > 0.05 for MSI-H, details Table 3).

When analysed in UICC Stage II/III only, similar effects on patient survival were noted. Tumour budding (*P* < 0.001 for all comparisons in MSS/MSI-H) and CRC subtypes (DSS: *P* < 0.001 in MSS; *P* = 0.003 in MSI-H/DFS: *P* < 0.001 in MSS; *P* = 0.009 in MSI-H) retained their prognostic impact MSS and MSI-H UICC Stage II/III CRCs, while WHO grade only remained significant in MSS UICC Stage II/III CRCs (DSS: *P* = 0.001/DFS: *P* < 0.001 in MSS; *P* > 0.05 for MSI-H in all comparisons, Fig. 2, Supplementary Fig. 4 and Table 4). In left-/right-sided CRCs as well as in the largest CRC subtype group (adenocarcinomas NOS), all parameters retained their strong prognostic impact (left-sided/right-sided CRCs/adenocarcinomas NOS only: *P* < 0.001 for all comparisons; data not shown). Notably, when analysed separately within CDX2-high vs CDX2-low/absent tumours, tumour budding/CRC subtypes remained highly prognostic (e.g. DFS: *P* < 0.001; data not shown) in all CDX2 groups, while WHO grade remained highly prognostic in CDX2-high (e.g. DFS: *P* < 0.001; data not shown), but not in CDX2-low/absent tumours.

### Multivariate analyses

In multivariate analyses (including age, gender, UICC stage, MSI-status, WHO grade, tumour budding, CRC subtypes and CDX2 groups) CDX2 expression was not an independent prognostic factor in the overall cohort (DSS: *P* = 0.97, 5; Table 5; DFS: *P* = 0.75, Supplementary Table 1; OS: *P* = 0.61, Supplementary Table 2) and in the subcohorts of UICC Stage II/III CRCs (DFS: *P* = 0.58; Supplementary Table 3***;*** DSS: *P* = 0.72, Supplementary Table 4; OS: *P* = 0.97, Supplementary Table 5) as well as in right-/left-sided CRCs (*P* > 0.05 for all comparisons, data not shown).

**Table 5:** Multivariate disease-specific survival analysis in the overall cohort under inclusion of CDX2 expression, age, gender CRC subtype, tumour budding, WHO grade, UICC stage and microsatellite status.

	HR (DSS)	Lower CI (95%)	Upper CI (95%)	*P* value
CDX2 subgroups				**0.97**
CDX2-high	1.00			
CDX2-low/absent	1.005	0.68	1.48	
WHO subtype				**0.03**
Adenocarcinoma NOS	1.00			
Mucinous adenocarcinoma	1.04	0.66	1.62	
Signet-ring cell carcinoma	0.92	0.32	2.65	
Medullary carcinoma	0.14	0.02	1.07	
Micropapillary adenocarcinoma	0.92	0.68	1.25	
Serrated adenocarcinoma	1.13	0.72	1.77	
Adenoma-like adenocarcinoma	0.73	0.17	3.02	
MANEC/NEC	2.84	1.08	7.47	
Tumour budding				**<0.001**
Bd1	1.00			
Bd2	3.27	2.31	4.63	
Bd3	6.90	4.73	10.07	
WHO grade				**0.01**
Low grade	1.00			
High grade	1.37	1.07	1.77	
UICC stage				**<0.001**
I	1.00			
II	1.70	1.01	2.87	
III	1.79	1.06	3.02	
IV	4.89	2.86	8.36	
Gender				**0.74**
Female	1.00			
Male	1.04	0.82	1.32	
Age group				**0.02**
Below median	1.00			
Median and above	1.33	1.04	1.69	
Microsatellite status				**0.85**
Microsatellite instable	1.00			
Microsatellite stable	1.04	0.66	1.66	

Bold values indicate statistical significance.

## Discussion

In this study, we evaluated the immunohistochemical expression of caudal-related homeobox transcription factor 2 (CDX2) in a large cohort of >1000 CRCs and correlated the results with staging parameters, microsatellite status and morphological parameters defined by the recent WHO classification (CRC subtypes, tumour budding, WHO grade). Finally, we analysed the prognostic relevance of CDX2 expression alone and compared it to the aforementioned histomorphologic parameters. Our study delivers three key messages: first, loss of CDX2 is of some prognostic relevance in CRC in univariate analyses, but its prognostic power is substantially inferior compared to the morphological factors defined by the WHO classification, especially when certain clinical subcohorts (e.g. UICC Stage II/III) are taken into account and vanishes when these parameters are incorporated into multivariate analyses. Second, CDX2 loss is massively enriched in MSI-H CRCs but fails to deliver prognostic information in this molecular subgroup. Third, loss of CDX2 is specifically enriched in certain CRC subtypes coming from both ends of the spectrum of biological aggressiveness, ranging from indolent variants such as medullary carcinomas to highly aggressive subtypes such as MANEC/NECs, arguing that both MSI-status, as well as the histomorphologic subtype of CRC, have to be considered before CDX2 might be used for clinical decision-making.

CDX2 is a highly conserved transcription factor that controls cell fate and differentiation in the intestinal epithelium and is diffusely expressed by non-neoplastic colonic epithelial cells [16]. CDX2 is also known to be strongly expressed by the majority of neoplasms of the colorectal system and is a marker that is frequently used in the daily practice of surgical pathology to verify or rule out an intestinal and especially colorectal origin of a carcinoma, for example in the setting of suspected CRC metastases in other organs or for histogenetic classification of cancers with unknown primary site [30]. For the diagnosis of primary CRC in resection specimen, CDX2 is not frequently used, because the vast majority of CRCs are accompanied by adenomatous precursor lesions that are diagnostic of primary CRC in combination with a morphologically compatible invasive carcinoma component. However, CDX2 gained increasing attention in the scientific community in recent years as some studies proposed a lost or diminished expression of CDX2 as an independent biomarker of a more aggressive disease course [17, 18, 21, 23, 26], while other studies could not generally confirm these results [31–37]. Compared to CDX2, which is normally assessed via immunohistochemistry, the recent WHO classification from 2019 defines the different histomorphological subtypes of CRC, tumour budding activity and the traditional WHO-grading algorithm in its essential diagnostic criteria for CRC, which represent purely histomorphological parameters that are assessed through the evaluation of HE-stained slides. In a recent study, we were able to confirm the high prognostic and in part stage-independent (CRC subtypes/tumour budding) prognostic impact of these parameters in the very same CRC cohort of >1000 tumours [8].

Generally confirming previous data, we observed significantly shorter survival parameters for CRC patients whose tumours showed a CDX2 loss in univariate analyses in our first general screening of the overall cohort. However, the prognostic power of CDX2 in this overall screening approach was considerably lower than those of the different CRC subtypes, WHO grade and tumour budding, which all showed stronger discrimination of survival groups than CDX2 expression, generally picturing the aforementioned WHO parameters as superior prognostic biomarkers in unselected CRC cohorts.

In the next step, we wanted to know if this general observation holds true when specific subcohorts of CRCs are investigated, which was the case when we analysed CRCs in UICC Stage II and III, where the prognostic power of CDX2 expression was also considerably lower compared to CRC subtypes, WHO grade and tumour budding. Although we can in principal confirm the results from previous studies such as the one from Dalerba et al. [17] that CDX2 is of some prognostic importance in univariate analyses in specific stage groups of CRC, our data for Stage II/III CRCs are comparable to the overall cohort, meaning that the purely morphological parameters allow for a significantly better assessment of different prognostic groups.

When we noticed the massive enrichment of CDX2 loss within MSI-H tumours, we decided to look into the prognostic relevance of all parameters within microsatellite subgroups of the overall cohort and Stage II/III cancers and observed that CDX2 has a considerable prognostic impact in MSS tumours, but shows no prognostic relevance in MSI-H CRCs, where tumour budding and the different CRC subtypes (but not WHO grade) showed a high prognostic significance. These findings are in line with results from recent studies [20, 25, 32, 35] and argue that the prognostic relevance of CDX2 expression is tightly connected to the microsatellite status.

Another interesting observation of our study is the dependence of the prognostic impact of CDX2 to tumour localisation. The distinction of right- vs. left-sided CRCs by itself showed no general prognostic impact in our cohort, which is in line with findings from a large scale dataset analysed in a SEER study [38] and other studies [39, 40]. However, there are conflicting results in the literature regarding the general prognostic relevance of tumour localisation of CRC as some authors propose an improved survival for left-sided CRCs [41, 42]. In right-sided CRCs (caecum to splenic flexure), we observed no prognostic impact of CDX2 expression in contrast to a retained high prognostic relevance of CRC subtypes, tumour budding and WHO grade comparable to the data from the overall cohort. In left-sided CRCs (Descending colon to rectum), however, we observed a—compared to the other subgroups—notably stronger prognostic impact of CDX2 loss on patient survival in univariate analyses, which was also present in left-sided microsatellite-instable CRCs, arguing that the evaluation of CDX2 expression has its highest relevance in left-sided colorectal carcinomas.

This general “sideness” of the prognostic impact of CDX2 expression may be related to the massive enrichment of CDX2-negative tumours in the medullary subtype of CRC, which is almost exclusively located in the right-sided colon and is associated with a comparatively benign clinical behaviour and thus probably narrows the potential worse prognostic effect of CDX2 loss in other CRC subtypes. Our findings argue towards the fact that not only the specific histologic subtype of CRC but also the tumour localisation and the microsatellite status have to be considered when the CDX2 status is assessed in order to determine patient prognosis.

In conclusion, our study confirms (but also significantly relativizes) the general prognostic relevance of CDX2 loss in colorectal cancer (in univariate analyses) and shows its association with tumour localisation, microsatellite status and certain CRC subtypes. Notably, CDX2 is not able to identify any additional subset of patients with a poor prognosis, that is not identified by either tumour budding, WHO grade or CRC subtypes. These central HE-based morphologic factors given by the WHO classification are generally prognostically superior compared to CDX2, with tumour budding being the strongest of the aforementioned parameters. These data suggest that these factors should be prioritised over CDX2 when histopathological parameters are used for clinical decision-making. The molecular mechanisms behind the enrichment of CDX2 loss in MSI-H tumours and rather benign (medullary CRC) or highly aggressive (MANEC/NEC) morphologic subtypes as well as in right-sided CRCs should be explored in further studies to potentially address potential therapeutic implications.

## Supplementary information

Supplementary material

aj_checklist

## Data Availability

All data relevant for this study are given with the main paper including figures, tables and Supplemental files. The tissue investigated for this study is archived in the Institute of Pathology of the Technical University of Munich.
