# Supplementary material for: Loss of CDX2 in colorectal cancer is associated with histopathologic subtypes and microsatellite instability but is prognostically inferior to hematoxylin–eosin-based morphologic parameters from the WHO classification
Source: Br J Cancer. 2021 Oct 6;125(12):1632–46. doi: 10.1038/s41416-021-01553-0 (PMC8651779; doi:10.1038/s41416-021-01553-0)
Supplement: Supplementary file 1 — Supplementary material [file 41416_2021_1553_MOESM1_ESM.pdf]

Suppl Figure 1

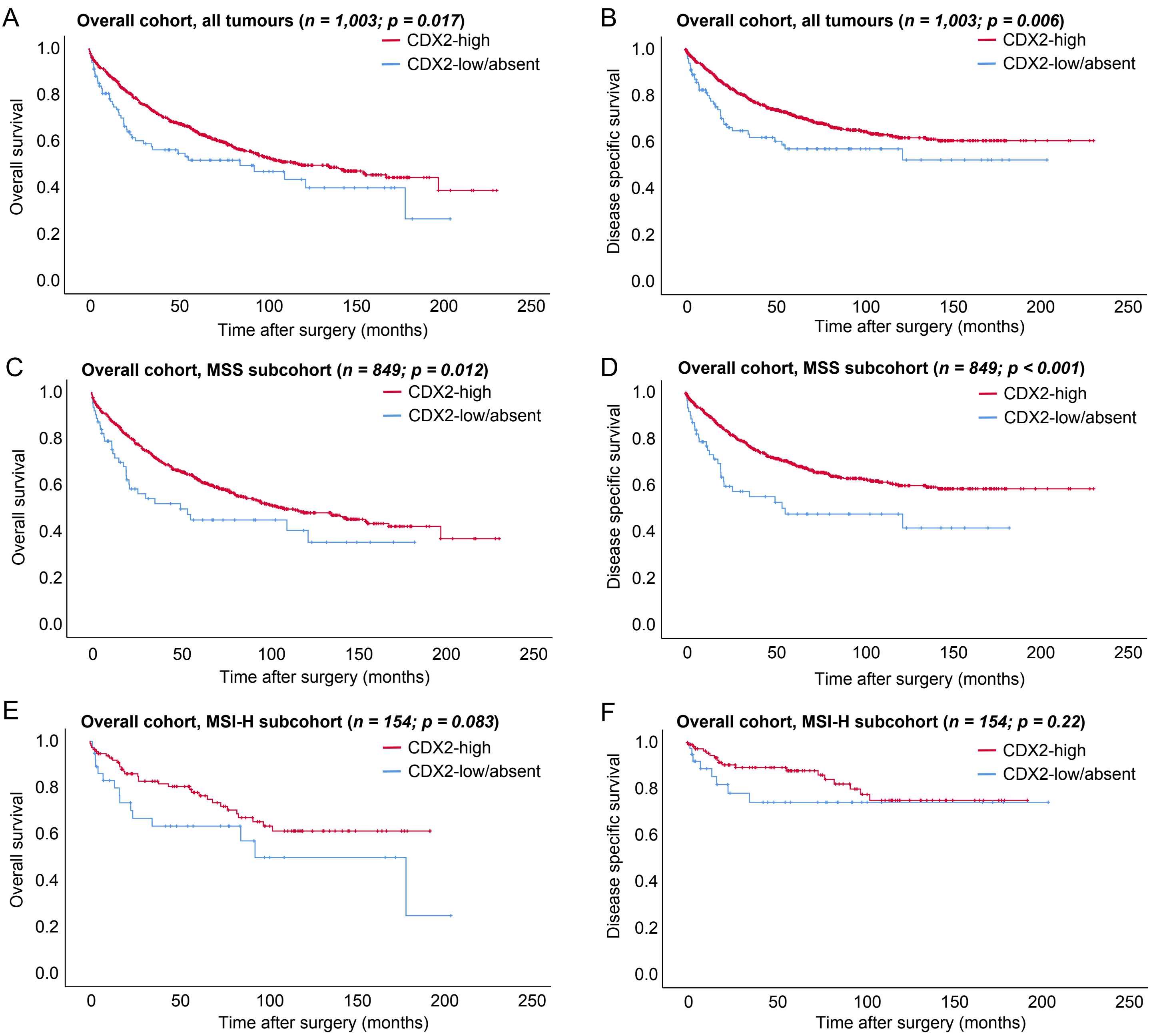

Suppl Figure 2

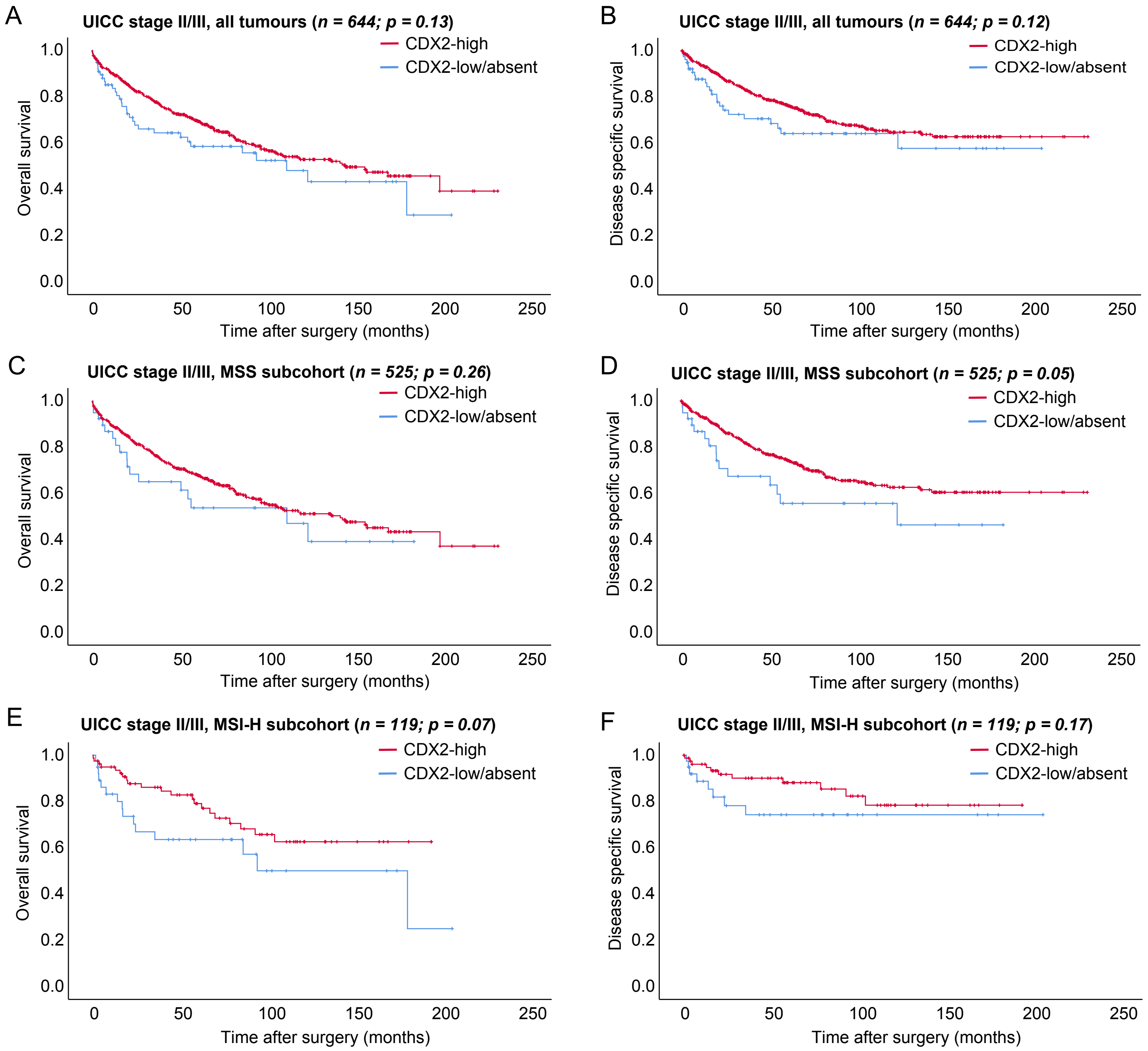

Suppl Figure 3

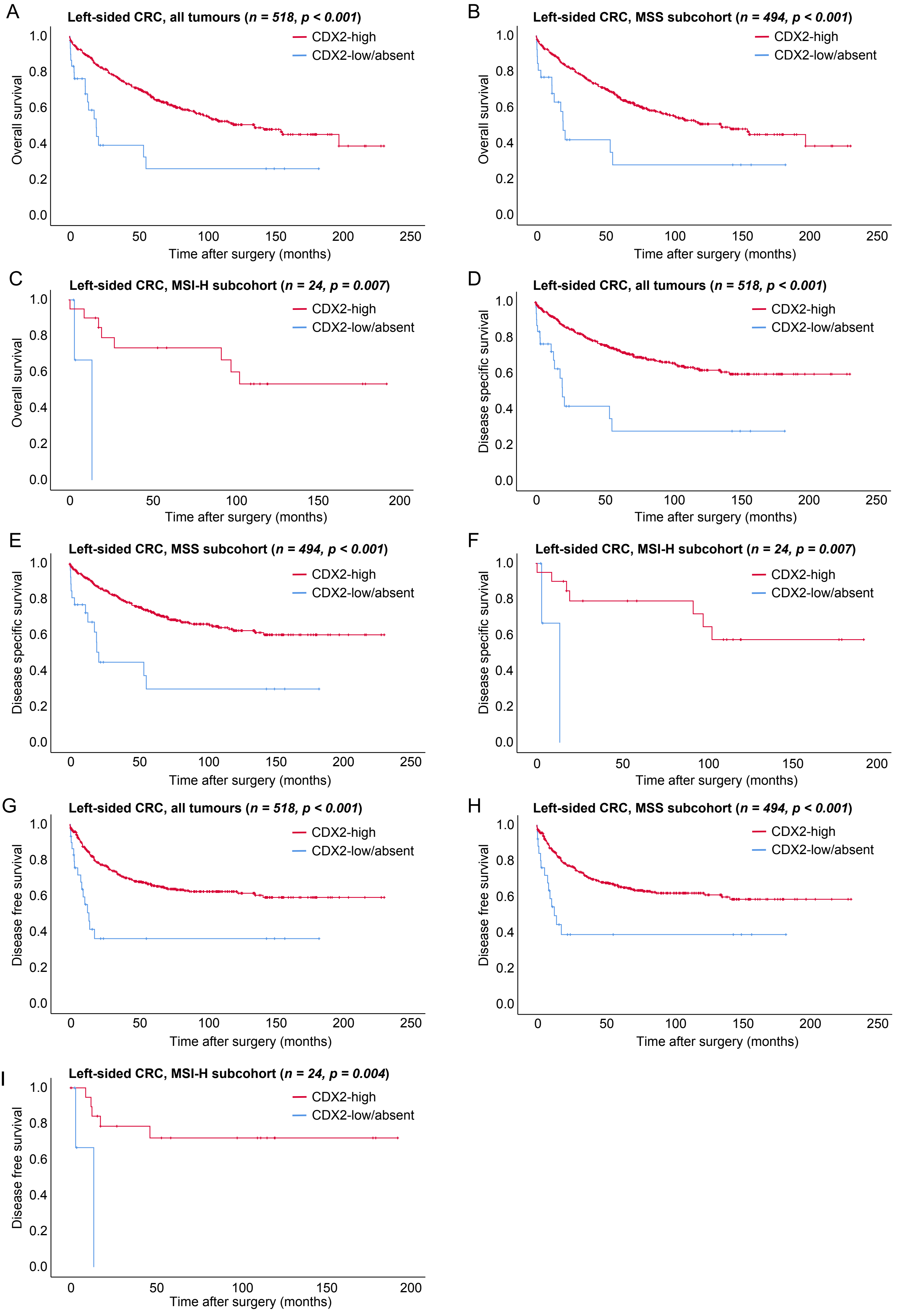

Suppl Figure 4

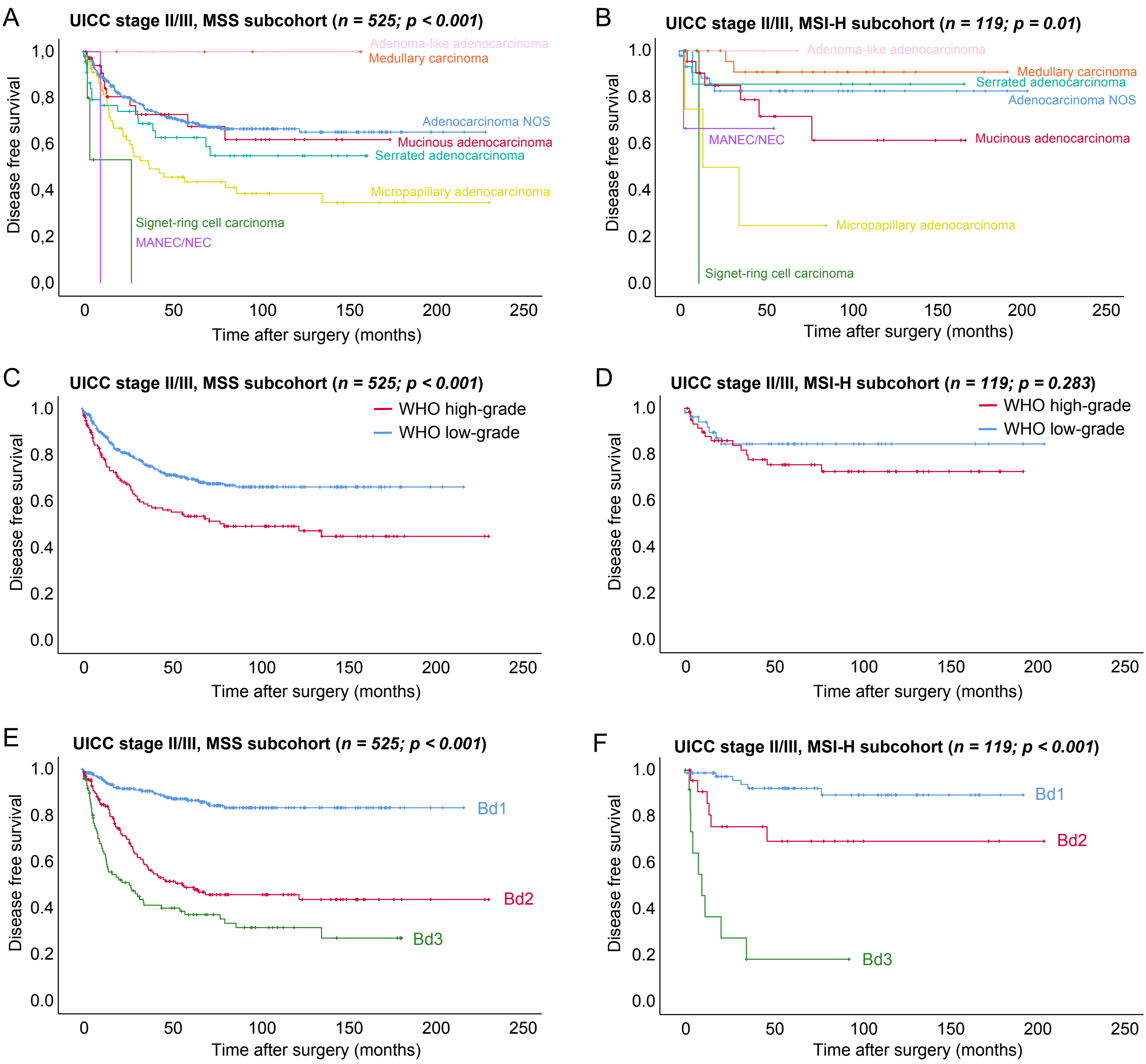

Supplementary Table 1		HR (DFS)	lower CI (95%)	upper CI (95%)	*p-value*
**CDX2 Subgroups**					***0.75***
	CDX2 high	1.00			
	CDX2 low/absent	1.06	0.74	1.53	
**WHO Subtype**					***0.07***
	Adenocarcinoma NOS	1.00			
	Mucinous adenocarcinoma	1.07	0.70	1.63	
	Signet-ring cell carcinoma	1.06	0.41	2.70	
	Medullary carcinoma	0.27	0.06	1.14	
	Micropapillary adenocarcinoma	0.88	0.65	1.19	
	Serrated adenocarcinoma	1.24	0.81	1.88	
	Adenoma-like adenocarcinoma	0.31	0.04	2.28	
	MANEC/NEC	1.68	0.59	4.82	
**Tumour Budding**					***<0.001***
	Bd1	1.00			
	Bd2	3.75	2.71	5.20	
	Bd3	7.17	5.00	10.27	
**WHO-grade**					***0.17***
	Low grade	1.00			
	High grade	1.19	0.93	1.51	
**UICC Stage**					***<0.001***
	I	1.00			
	II	1.36	0.84	2.22	
	III	2.06	1.29	3.30	
	IV	4.25	2.95	6.96	
**Gender**					***0.92***
	female	1.00			
	male	1.01	0.80	1.27	
**Age group**					***0.62***
	Below median	1.00			
	Median and above	0.94	0.75	1.19	
**Microsatellite status**					***0.96***
	Microsatellite stable	1.00			
	Microsatellite instable	0.99	0.64	1.53	

Supplementary Table 2		HR (OS)	lower CI (95%)	upper CI (95%)	*p-value*
**CDX2 Subgroups**					***0.61***
	CDX2 high	1.00			
	CDX2 Low/absent	0.92	0.65	1.29	
**WHO Subtype**					***0.04***
	Adenocarcinoma NOS	1.00			
	Mucinous adenocarcinoma	1.09	0.75	1.60	
	Signet-ring cell carcinoma	0.78	0.28	2.19	
	Medullary carcinoma	0.72	0.33	1.58	
	Micropapillary adenocarcinoma	0.82	0.62	1.09	
	Serrated adenocarcinoma	1.11	0.77	1.61	
	Adenoma-like adenocarcinoma	1.39	0.72	2.70	
	MANEC/NEC	2.67	1.03	6.89	
**Tumour Budding**					***<0.001***
	Bd1	1.00			
	Bd2	2.26	1.74	2.95	
	Bd3	4.52	3.34	6.11	
**WHO-grade**					***0.01***
	Low grade	1.00			
	High grade	1.33	1.07	1.65	
**UICC Stage**					***<0.001***
	I	1.00			
	II	1.18	0.84	1.65	
	III	1.07	0.76	1.52	
	IV	2.76	1.90	4.00	
**Gender**					***0.1***
	female	1.00			
	male	1.19	0.97	1.46	
**Age group**					***<0.001***
	Below median	1.00			
	Median and above	1.65	1.35	2.03	
**Microsatellite status**					***0.73***
	Microsatellite instable	1.00			
	Microsatellite stable	1.05	0.73	1.50	

**Supplementary Table 3**	**HR (DFS)**	**lower CI (95%)**	**upper CI (95%)**	***p-value***
**CDX2 Subgroups**				**0.58**
CDX2 high	1.00			
CDX2 Low/absent	1.14	0.72	1.81	
**WHO Subtype**				***0.08***
Adenocarcinoma NOS	1.00			
Mucinous adenocarcinoma	1.58	0.89	2.82	
Signet-ring cell carcinoma	2.54	0.88	7.36	
Medullary carcinoma	0.33	0.08	1.47	
Micropapillary adenocarcinoma	1.08	0.73	1.60	
Serrated adenocarcinoma	1.50	0.89	2.52	
Adenoma-like adenocarcinoma	0.10	0.1	>200	
MANEC/NEC	1.90	0.43	8.34	
**Tumour Budding**				**<0.001**
Bd1	1.00			
Bd2	4.24	2.86	6.30	
Bd3	7.32	4.66	11.51	
**WHO-grade**				**0.17**
Low grade	1.00			
High grade	1.25	0.91	1.71	
**Gender**				***0.92***
female	1.00			
male	1.11	0.82	1.50	
**Age group**				**0.52**
Below median	1.00			
Median and above	0.91	0.67	1.23	
**Microsatellite status**				***0.29***
Microsatellite instable	1.00			
Microsatellite stable	1.34	0.78	2.29	

Supplementary Table 4	HR (DSS)	lower CI (95%)	upper CI (95%)	*p-value*
**CDX2 Subgroups**				**0.72**
CDX2 high	1.00			
CDX2 low/absent	1.09	0.66	1.81	
**WHO Subtype**				***0.06***
Adenocarcinoma NOS	1.00			
Mucinous adenocarcinoma	1.23	0.66	2.29	
Signet-ring cell carcinoma	2.40	0.71	8.11	
Medullary carcinoma	0.15	0.02	1.13	
Micropapillary adenocarcinoma	0.91	0.59	1.39	
Serrated adenocarcinoma	1.03	0.60	1.78	
Adenoma-like adenocarcinoma	0.10	0.10	>200	
MANEC/NEC	2.16	0.48	9.61	
**Tumour Budding**				**<0.001**
Bd1	1.00			
Bd2	3.43	2.25	5.21	
Bd3	6.45	4.03	10.33	
**WHO-grade**				**0.01**
Low grade	1.00			
High grade	1.35	0.96	1.88	
**Gender**				***0.96***
female	1.00			
male	0.99	0.72	1.37	
**Age group**				**0.009**
Below median	1.00			
Median and above	1.55	1.12	2.16	
**Microsatellite status**				***0.39***
Microsatellite instable	1.00			
Microsatellite stable	1.27	0.73	2.21	

**Supplementary Table 5**	**HR (OS)**	**lower CI (95%)**	**upper CI (95%)**	***p-value***
**CDX2 Subgroups**				**0.97**
CDX2 high	1.00			
CDX2 low/absent	0.99	0.65	1.53	
**WHO Subtype**				***0.21***
Adenocarcinoma NOS	1.00			
Mucinous adenocarcinoma	1.30	0.80	2.12	
Signet-ring cell carcinoma	1.89	0.57	6.28	
Medullary carcinoma	0.72	0.32	1.63	
Micropapillary adenocarcinoma	0.78	0.52	1.16	
Serrated adenocarcinoma	0.99	0.63	1.56	
Adenoma-like adenocarcinoma	1.00	0.24	4.12	
MANEC/NEC	2.02	0.46	8.76	
**Tumour Budding**				**<0.001**
Bd1	1.00			
Bd2	2.34	1.70	3.21	
Bd3	3.99	2.72	5.83	
**WHO-grade**				**0.16**
Low grade	1.00			
High grade	1.23	0.92	1.64	
**Gender**				***0.33***
female	1.00			
male	0.87	0.66	1.15	
**Age group**				**<0.001**
Below median	1.00			
Median and above	1.98	1.50	2.62	
**Microsatellite status**				***0.73***
Microsatellite instable	1.00			
Microsatellite stable	1.18	0.77	1.81	

Legends

Supplementary Figure 1: Prognostic relevance of CDX2 expression groups on Overall and Disease Free Survival in the overall cohort including microsatellite subgroups.

Supplementary Figure 2: Prognostic relevance of CDX2 expression groups on Overall and Disease Free Survival in the UICC stage II/III cohort including microsatellite subgroups.

Supplementary Figure 3: Prognostic impact of CDX2 in all left-sided CRCs including Microsatellite subgroups.

Supplementary Figure 4: Prognostic relevance on Disease Free Survival of the central HE based morphologic factors CRC subtypes, WHO-grade and Tumour Budding in UICC stage II/III microsatellite subgroups.

Supplementary Table 1: Multivariate disease free survival analysis in the overall cohort under inclusion of CDX2 expression, age, gender CRC subtype, Tumour Budding, WHO-grade, UICC stage and microsatellite status.

Supplementary Table 2: Multivariate overall survival analysis in the overall cohort under inclusion of CDX2 expression, age, gender CRC subtype, Tumour Budding, WHO-grade, UICC stage and microsatellite status.

Supplementary Table 3: Multivariate disease free survival analysis in UICC stage II/III CRCs under inclusion of CDX2 expression, age, gender CRC subtype, Tumour Budding, WHO-grade and microsatellite status.

Supplementary Table 4: Multivariate disease specific survival analysis in UICC stage II/III CRCs under inclusion of CDX2 expression, age, gender CRC subtype, Tumour Budding, WHO-grade and microsatellite status.

Supplementary Table 5: Multivariate overall survival analysis in UICC stage II/III CRCs under inclusion of CDX2 expression, age, gender CRC subtype, Tumour Budding, WHO-grade and microsatellite status.
